# Estimating Point and Interval Frequency of Antigen-Specific CD4^+^ T Cells Based on Short In Vitro Expansion and Improved Poisson Distribution Analysis

**DOI:** 10.1371/journal.pone.0042340

**Published:** 2012-08-07

**Authors:** Giulia Di Lullo, Francesca Ieva, Renato Longhi, Anna Maria Paganoni, Maria Pia Protti

**Affiliations:** 1 Tumor Immunology Unit, San Raffaele Scientific Institute, Milan, Italy; 2 Division of Immunology, Transplantation and Infectious Diseases, San Raffaele Scientific Institute, Milan, Italy; 3 Laboratorty for Modeling and Scientific Computing (MOX), Dipartimento di Matematica, Politecnico di Milano, Milan, Italy; 4 Consiglio Nazionale delle Ricerche (CNR), Istituto di Chimica del Riconoscimento Molecolare, Milan, Italy; Virginia Tech, United States of America

## Abstract

**Background:**

Knowledge of antigen-specific CD4^+^ T cells frequencies is pivotal to the choice of the antigen to be used in anti-viral and anti-tumor vaccination procedures and for monitoring of immune responses. Methods that employ small cell numbers from patient samples, are easy to perform and do not require complex techniques/instrumentations and therefore standardization are desirable.

**Methodology/Principal Findings:**

Purified blood CD4^+^ T cells from healthy donors were cultured with autologous antigen presenting cells in several replicate wells in equal numbers in the absence (un-stimulated wells) or in the presence of synthetic peptides corresponding to viral antigens promiscuous HLA-DR epitopes (antigen-stimulated wells). At day 7 of culture low dose IL-2 was added and at day 14 IFN-γ and IL-5 release in the supernatant was measured. A statistical analysis approach, based on Poisson distribution, was then implemented to calculate the frequency of viral-specific CD4^+^ T cells. We first determined a patient-specific exceptionality threshold of cytokine release in the un-stimulated wells and then, based on this threshold, we counted the inactive/active wells within the antigen-stimulated wells. This number, along with the number of cells per well, allowed the point and interval estimates of frequencies. A ready-to-use Excel worksheet template with automatic calculations for frequencies estimate was developed and is provided as a supplemental file (Table S9).

**Conclusions/Significance:**

We report a simple experimental procedure combining short term *in vitro* cell culture with statistical analysis to calculate the frequency of antigen-specific CD4^+^ T cells. The detailed experimental procedure along with the Excel applicative are a valuable tool for monitoring immune responses in the clinical practice.

## Introduction

Ag-specific CD4^+^ T cells play an important role in induction and regulation of anti-viral and anti-tumor immunity [1], [2], [3], [4]. In the last years preventive and therapeutic vaccination strategies using viral and tumor antigens (Ags) have been developed aiming at activation of naïve or expansion of spontaneous viral and tumor Ag-specific memory CD4^+^ T cells; leading to the first FDA approved therapeutic antitumor vaccine [5].

A fundamental requisite for clinical efficacy of anti-viral and anti-tumor vaccines is the induction/expansion of Ag-specific CD4^+^ T cells; therefore, pre- and post- vaccination immune monitoring should evaluate and compare the presence, frequency, phenotype and function of Ag-specific CD4^+^ T cells. Furthermore, monitoring of spontaneous Ag-specific CD4^+^ T cell responses prior to vaccination is also instrumental to the choice of the immunogen to be used [6].

Different methods to detect viral and tumor Ag-specific CD4^+^ T cells in healthy carriers or infected individuals and neoplastic patients are being used (*i.e*., ELISPOT assay, intracellular cytokine detection by flow cytometry, proliferation and cytokine release assays, tetramer staining and limiting dilution assay) with different pros and cons [7], [8], [9], [10], [11], [12], [13], [14]. Therefore, it is important to tailor the method to use for immune-monitoring purposes by taking into consideration the specific vaccination strategy and the clinical setting you need to monitor.

An important issue to consider when the different methods are compared is the possibility to detect Ag-specific CD4^+^ T cells directly *ex-vivo*, because of the possible biases derived from cell culture. However, spontaneous Ag-specific CD4^+^ T cells are present at very low frequencies in peripheral blood [15] and *in vitro* expansion is usually needed to allow their detection. Nonetheless, culture conditions should avoid excessive manipulation with multiple re-stimulations *in vitro* with the relevant Ags to better preserve the *in vivo* Ag-specific CD4^+^ T cell functional characteristics. Moreover, best characteristics for a large scale immune monitoring approach in a clinical setting should be on the one hand feasible with small cell samples, no cumbersome, no expensive and with no need of a sophisticated and difficult to standardize instrumentation and on the other hand to be the most informative such as able to detect both the frequency and possibly multiple functions of Ag-specific memory CD4^+^ T cells.

In the present study we describe a short-term *in vitro* re-stimulation culture method combined with an *ad hoc* statistical analysis for the calculation of the frequency of Ag-specific memory CD4^+^ T cells. To this aim first we implemented a culture method previously set for detection of the presence and quality of spontaneous viral and tumor Ag-specific CD4^+^ T cells in the blood of healthy individuals and neoplastic patients [16], [17], [18], [19]. Second, we developed an improved statistical analysis based on the Poisson distribution that allowed us to set the calculation for the estimate of point and interval frequencies (*i.e*., the quantity) of Ag-specific CD4^+^ T cells.

## Materials and Methods

### Donors and Antigens

Peripheral blood mononuclear cells (PBMCs) were obtained from the blood of 17 healthy donors. The Institutional Ethics Committee (Comitato Etico Fondazione Centro San Raffaele del Monte Tabor, Istituto Scientifico Ospedale San Raffaele) had approved the study protocol and written informed consent was obtained from all donors before blood sampling. Sequence 307–319 from influenza hemagglutinin (HA) (HA_307–319_) and sequence 280–294 from Epstein-Barr nuclear antigen (Ag) 2 (EBNA2)(EBNA2_280–294_) were selected as highly promiscuous HLA-DR binders (*i.e.*, able to bind to several alleles) [20] to be used for recognition by CD4^+^ T cells from HLA-DR unselected donors (*i.e*., bearing disparate HLA-DR alleles). Synthetic peptides corresponding to the selected sequences were synthesized by the stepwise solid-phase method as described previously [21]. The peptides were lyophilized, reconstituted in DMSO at 10 mg/ml, and diluted in RPMI 1640 (BioWhittaker) to the final concentration of 10 µg/ml. Keyhole limpet hemocyanin (KLH) (Calbiochem) was used at 10 µg/ml.

### Short-term Cultures of Ag-specific CD4^+^ T Cells

#### Culture for identification of responsive donors

The culture conditions were as previously described [16], [18], [19] with slight modifications. Briefly, frozen CD4^+^ T cells purified from PBMCs by magnetic beads (Miltenyi Biotec) were thawed and cultured in 96-well round-bottom plates at 1×10^5^/well in six replicates for each condition in 200 µl/well of tissue culture medium, composed by X-VIVO 15 (BioWhittaker) supplemented with penicillin (100 U/ml; BioWhittaker), streptomycin (50 mg/ml; BioWhittaker) and 3% heat-inactivated pooled human serum (BioWhittaker), in the presence of irradiated CD4^+^ T cell-depleted PBMCs as antigen presenting cells (APCs), at a CD4^+^ T cell : APC ratio of 1∶3. In some experiments, as detailed in the Results, cells were plated at 1–3×10^4^/well. Stimuli were: phytohemagglutinin (PHA) (10 µg/ml; Sigma-Aldrich), as positive control; KLH (10 µg/ml) and viral peptides (HA_307–319_ and EBNA2_280–294_)(10 µg/ml). CD4^+^ T cells in the presence of the APCs only were used as baseline (un-stimulated). At day 7, half medium from each well was removed and replenished with fresh medium containing IL-2 (25 IU/ml; R&D Systems) without further Ag stimulation. At day 14, 100 µl of supernatant collected from each well of the six replicates for each condition were pooled (6-wells pool) and used for detection of IFN-γ and IL-5 by ELISA (Mabtech), according to the manufacturers’ instructions. Samples from Ag-stimulated CD4^+^ T cells (pooled supernatants of stimulated-wells) were considered positive if at least double of the values from un-stimulated cells (pooled supernatants of un-stimulated wells) and above 50 pg/ml. For proliferation assay single wells were pulsed for 16 hours with ^[3H]^TdR (1 mCi per well, 6.7 Ci/mol; Amersham Corp.) and cells collected with a FilterMate Universal Harvester (Packard Instruments) in specific plates (Unifilter GF/C; Packard). Incorporated thymidine was measured in a liquid scintillation counter (TopCount NXT; Packard).

#### Culture for calculation of the frequency of Ag-specific CD4^+^ T cells

Culture conditions were similar to those described above with slight modification. Specifically, the total number of cells tested per each condition (− and + Ag) was 9×10^5^, 3×10^4^/well for a total of 30 wells. In one experiment (as indicated in the Results), total cells seeded were 5×10^5^, 1×10^4^/well for a total of 50 wells. IFN-γ and IL-5 release was detected by ELISA as described above but on single wells.

#### CD4^+^ T cell stimulation assays for Ag specificity

Peptide-stimulated CD4^+^ T cells at day 21 of culture were collected, washed and re-plated (1×10^4^/well) in triplicate in 96-well-round-bottom plates in the presence of irradiated autologous PBMCs (1∶5), as APCs, and the relevant peptide (10 µg/ml). Three wells with CD4^+^ T cells plus APCs did not receive any stimulus to determine the basal condition. After 48 hours, IFN-γ and IL-5 release was measured in the culture supernatant by ELISA as described above.

### Statistical Analysis

#### Exceptionality threshold

We first studied the distribution of the cytokines concentration in the un-stimulated wells in order to detect and exclude possible outliers and then to compute a threshold beyond which an observation would result from such a distribution with a very low probability (say *ε*). Once the threshold has been established, if the cytokines concentration in an Ag-stimulated well is over such a threshold we conclude that the well is activated. In order to compute this exceptionality threshold we used the Cantelli’s inequality [22].

#### Estimate of frequency of Ag-specific CD4^+^ T cells

We based our analysis on the assumption that the number *M* of activated cells in each well containing *N* cells follows a Poisson distribution with parameter 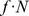 where *f* is the frequency of Ag-specific cells that we want to estimate. The data available to estimate the frequency *f* are observations by a Bernoulli variable, which is equal to 1 if the well is declared inactive (using the procedure proposed above) and 0 if the well is declared active. The probability for a well to be inactive is equal to the probability that *M* = 0 and so is 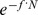. Therefore, we can model the activation data of the wells as observations from a sample of independent and identically distributed Bernoulli variables Y_i_ with parameter 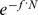.

#### Point estimation

The maximum likelihood estimation of *f* is equal to


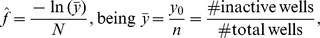


where y_0_ is the number of inactive wells, *n* is the number of wells and *N* the number of cells in each well.

#### Interval estimation

We used the Clopper-Pearson [23] confidence interval for an unknown proportion p: the confidence interval of level (1-α), when y_0_ = 1, 2, …, n-1 is


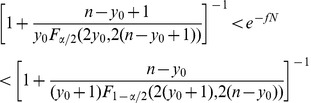
(1)

where *F_c_* (*a, b*) is the quantile of order *c* for a Fisher distribution with (*a, b*) degrees of freedom. From equation (1) we can deduce the confidence interval of level (1-α) for *f*:


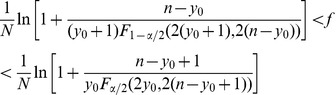
(2)

When *y_0_* =  n or *y_0_* =  0 the previous confidence interval in (1) becomes meaningless and in these extreme cases we can use the rule of three (see [24] for more details) to construct a 95% confidence level for f:


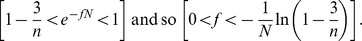


This means that when *y_0_* =  *n* (all wells are inactive), the point estimation of the frequency of Ag-specific CD4^+^ T cells is zero, but in a number of independent observations (*i.e.*, experiments), we expect that in 95% cases the frequency could range up to the upper extreme of the confidence interval. Analogously, when all cultures result active (*y_0_ = 0*), the 95% confidence interval for *f*, constructed using *the rule of three* becomes:


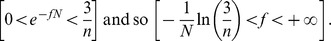


## Results

### Setting Culture Conditions for the Study of Ag-specific Memory CD4^+^ T Cell Responses

We took advantage of the *ex-vivo* re-stimulation assay developed in our laboratory to test the presence and the quality (*i.e.*, the cytokine profile) of anti-viral and anti-tumor recall CD4^+^ T cell responses in healthy donors and neoplastic patients [16], [18], [19]. In this assay 3×10^4^ purified CD4^+^ T cells are cultured with autologous APCs in six replicates for each condition in the absence and in the presence of relevant peptide(s) for a week; at day 7, half of the medium from each well is removed and replenished with fresh medium containing low doses IL-2. At day 14, cytokine release in the supernatant of the six pooled wells for each condition and ^3^H-thymidine incorporation for single well are measured. For cytokine release assays, samples from peptide-stimulated wells are considered positive if at least double of the values from un-stimulated wells and above 50 pg/ml. For the ^3^H-thymidine incorporation assays, responses are considered positive when significantly higher than the un-stimulated wells (*i.e.*, CD4+APC), as determined by unpaired, one-tailed Student’s *t* test. As before day 14 usually neither cytokine release nor proliferation are detectable, we assumed that the assay allows the expansion of *in vivo* activated, low frequency memory CD4^+^ T cells. This assumption was supported by the lack of reactivity of cord blood naïve CD4^+^ T cells to peptides corresponding to sequences of the E6 and E7 proteins of the human papilloma virus type-18, which we otherwise showed to be immunogenic in infected healthy individuals and cervical cancer patients [16], [17].

**Figure 1:**
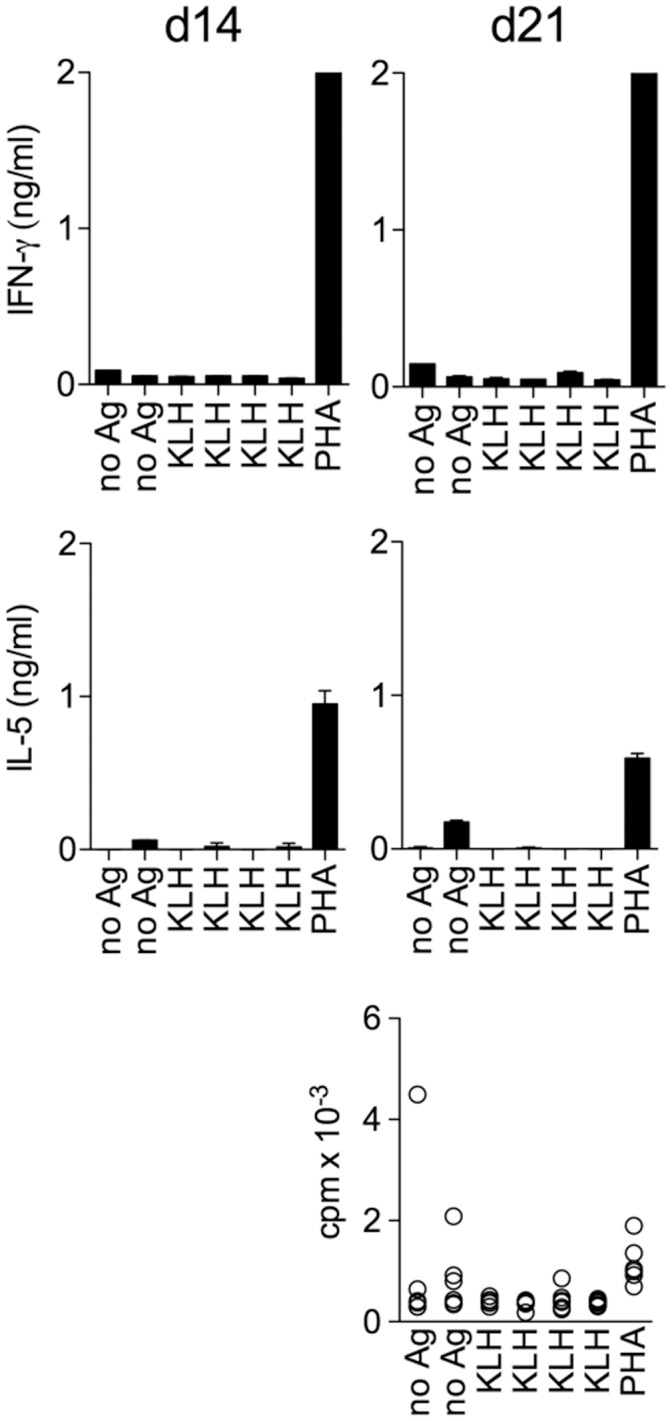
The *in vitro* re-stimulation culture used does not induce priming of naïve CD4^+^ T cells. Total CD4^+^ T cells were purified from the blood of adult healthy donors and put in culture (1×10^5^/well) in several replicates in the absence (no Ag = CD4+APCs) or in the presence of the primary Ag KHL. PHA stimulation was used as positive control. At days 14 (d14) and 21 (d21) supernatants were collected and groups of six wells were pooled and IFN-γ and IL-5 release quantified by ELISA. For ^3^H-thymidine incorporation assays data from each well are shown.

In the present study, to definitely exclude that our culture system induces *in vitro* priming of T cell responses, CD4^+^ T cells purified from the blood of 7 adult healthy donors were stimulated with the primary antigen KLH. To increase the possibility to detect KLH specific CD4^+^ T cells we increased the number of total plated cells to 2.4–4.8×10^6^ cells (1×10^5^/well in several replicate wells). This number was chosen large enough in order to be confident to possibly detect Ag-specific naïve CD4^+^ T cells, which have been recently demonstrated to range in few to 70 per million CD4^+^ naïve T cells [25], [26]. We found that none of the donors showed KLH-specific CD4^+^ T cell reactivity in terms of both cytokine release (IFN-γ and IL-5) and proliferation (Fig. 1); even when cultures were prolonged till day 21 to increase the chances to expand more rare precursors.

**Figure 2:**
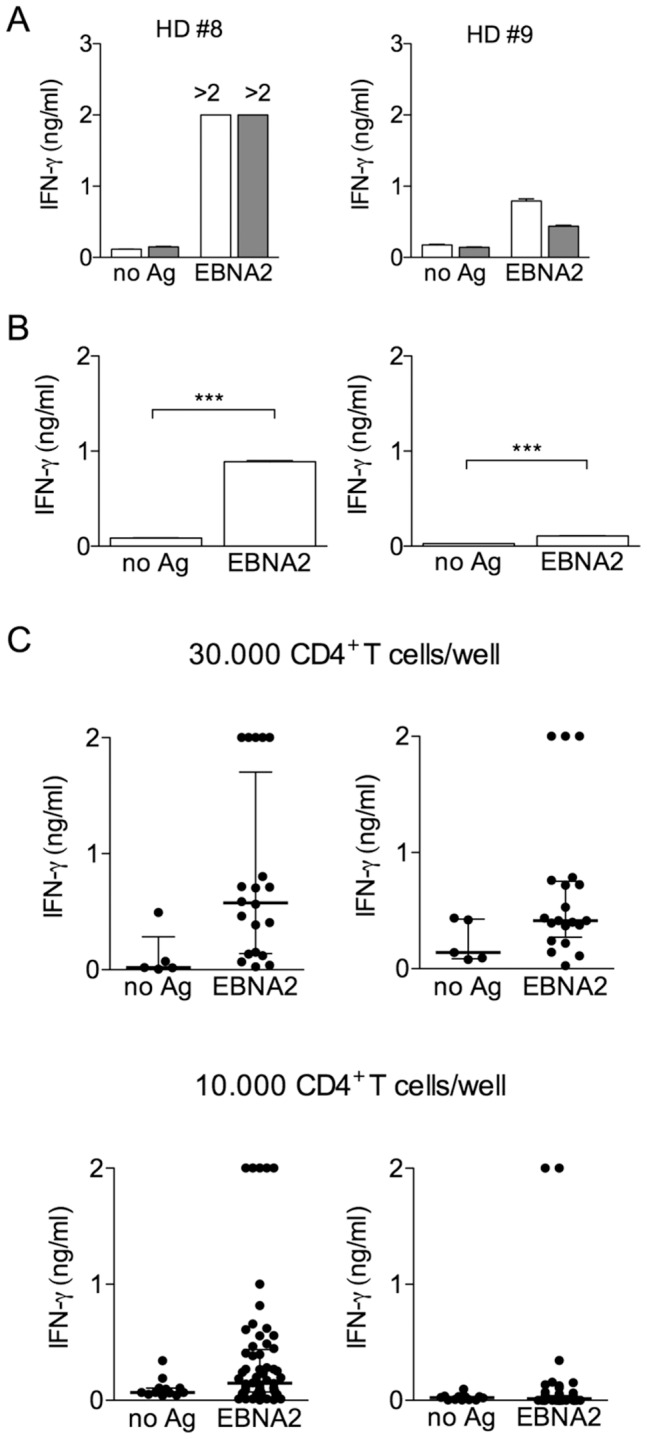
The amount of IFN-γ released by CD4^+^ T cells correlates with the number of positive wells. Healthy donors (HD) #8 and #9 were chosen as representative for donors with different amplitude of IFN-γ release by viral Ag-stimulated CD4^+^ T cells. (A) 1×10^5^/well CD4^+^ T cells were cultured with autologous APCs in the absence (6 wells) and in the presence (6 wells) of the EBNA2 peptide. IFN-γ release was measured at days 14 (white columns) and 21 (grey columns) of culture on supernatant pools of the 6 wells. Values are the means of duplicate determinations ± SEM. (B) After a further week of culture (d21), EBNA2-stimulated CD4^+^ T cells were collected, washed and tested for EBNA2-specific recognition by challenge with un-pulsed or peptide-pulsed autologous APCs and IFN-γ specific release was measured after 48 hours. Values are the means of triplicate determinations ± SEM. Responses significantly higher than the blanks (*i.e.*, no Ag  =  CD4+APC) are indicated as ***p<0,001 (determined by unpaired, one-tailed Student’s *t* test). (C) CD4^+^ T cells from the same two donors were cultured plating two seeding densities, *i.e.* 3×10^4^ cells/well (5 un-stimulated wells and 20-stimulated wells) (upper panels) and 1×10^4^ cells/well (10 un-stimulated wells and 60 stimulated wells)(lower panels). At day 14, IFN-γ secretion was measured as above but on single well supernatants. Dots are means of duplicate determinations.

After establishing that our culture system does not induce *in vitro* priming of naïve CD4^+^ T cells, we screened 8 healthy donors to identify suitable donors for further analysis of Ag-specific T cell frequency. As Ags we chose two peptides corresponding to the promiscuous viral sequences HA_307–319_ and EBNA2_280–294_ (from now on referred as HA and EBNA2, respectively) because we previously found that these peptides were recognized by up to almost 80% of subjects tested, independently of the HLA-DR alleles carried [19]. We found HA and/or EBNA2 specific CD4^+^ T cells in all but one donor, although the level of cytokine released varied among them. As an example, in Fig. 2A, we report the results obtained with donors #8 and #9 displaying clearly different levels of IFN-γ release in the presence of the EBNA2 peptide. To prove that in both cases we were measuring Ag-specific CD4^+^ T cells we perform a specificity assay. CD4^+^ T cells stimulated with EBNA2 were collected, washed, and tested for IFN-γ release in the absence or in the presence of the peptide (Fig. 2B). In both cases IFN-γ release was significantly higher in the presence than in the absence of the peptide, demonstrating the EBNA2 specificity, and the level of cytokine released mirrored the differential amplitude of response between the two donors already detected in the supernatant of day 14 cultures. We reasoned that one possible reason for the variable magnitude of cytokine release among donors could be the presence and expansion of a different number of EBNA2-specific CD4^+^ T cells originally present in the cultures. In this case, the 14-day culture system could be exploited for the calculation of Ag-specific CD4^+^ T cell frequency by application of the Poisson distribution, considering that at least one Ag-specific CD4^+^ T cell should be present in each positive well. Thus, we repeated the culture from the same two donors maintaining the total number of cells tested (6×10^5^) but with two different concentrations of cells/well (*i.e.*, 1×10^4^ and 3×10^4^, respectively) and performed single-well INF-γ detection. Five and ten wells per conditions, respectively, were left un-stimulated to determine the background level of cytokine release. Ag-stimulated wells were considered positive if IFN-γ release was 2-fold higher than the mean value of the un-stimulated wells. In both cell-plating densities the number of positive wells within the Ag-stimulated was higher for donor #8 (14 and 25, respectively) than donor #9 (8 and 12, respectively)(Fig. 2C). We concluded that indeed our culture system could be exploited also for frequency estimation.

### Statistical Basis and Experimental Validation for the Analysis of CD4^+^ T cell Frequencies

**Figure 3:**
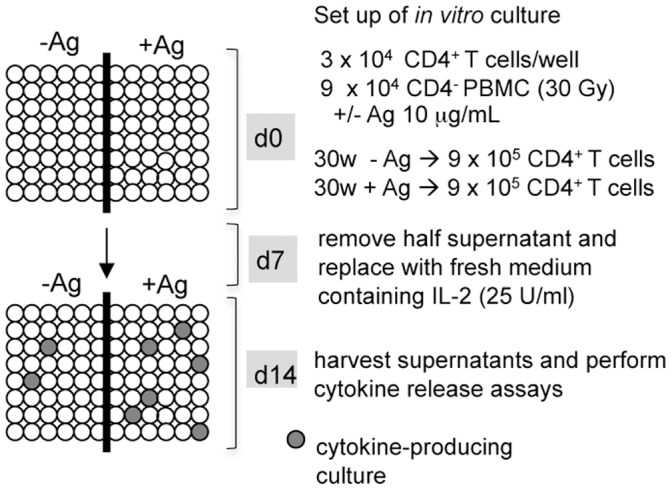
Schematic representation of the culture conditions set for estimate of Ag-specific CD4^+^ T cells frequency. CD4^+^ T cells were purified from total PBMCs and seeded in 96-well plates at 3×10^4^/well in the presence of irradiated autologous APCs (1∶3 ratio) for a total of 0.9×10^6^ CD4^+^ T cells for each condition, *i.e*., in the absence (−Ag) or in the presence (+Ag) of the relevant peptide. At day 7, low dose IL-2 was added without further Ag stimulation. At day 14, single-well cytokine release (IFN-γ and IL-5) was measured by ELISA.

A major concern when applying the culture system described above is the identification of a threshold of cytokine release by un-stimulated cells that would guarantee discriminating between Ag-specific and non Ag-specific CD4^+^ T cells activation and function. Thus, we decided to further modify the experimental procedure (Fig. 3) by enlarging the number of un-stimulated wells, on which to perform statistical analysis to set a proper threshold of unspecific cytokine release. A total of 18×10^5^ CD4^+^ T cells from 7 donors were seeded in 60 wells at 3×10^4^/well. Half wells were left un-stimulated (−Ag) and half were stimulated with the relevant peptide(s)(+Ag). IFN-γ and IL-5 release was assayed at day 14 of culture in each independent well. Data of cytokines release for each donor are reported in Tables S1, S2, S3, S4, S5, S6, S7.

#### Outlier exclusion and exceptionality threshold in the un-stimulated wells

The basal activation state of CD4^+^ T cells in the absence of peptide-specific stimulation is extremely variable among donors (Tables S1, S2, S3, S4, S5, S6, S7), therefore criteria to define donor-specific thresholds must be defined. We studied the distribution of cytokines concentration in the un-stimulated wells in order to detect and exclude possible outliers and to set a threshold for single-well cytokines release to be used to discriminate between un-specific and Ag-stimulated cultures.

We considered as outliers all data over the value Q3+1.5*(Q3-Q1), where Q1 is the quantile of order 25% and Q3 the quantile of order 75% of the distribution of un-stimulated wells cytokines concentration and excluded these data in the following analyses. We then computed an exceptionality threshold, based on the Cantelli’s inequality, beyond which an observation would belong to the distribution of the un-stimulated wells with a very low (*i.e.*, exceptional) probability (*ε*). The Cantelli’s inequality states that for a random variable X, with mean *µ* and standard deviation *σ*, for every *κ>0* we have:


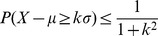


In particular, if we set


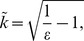
 the previous inequality becomes


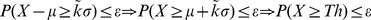


where 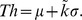 *Th* is the exceptionality threshold and µ and *σ* are the mean and the standard deviation of the X distribution. In our case we estimated *µ* and *σ* using the values of the cytokines concentration of the un-stimulated wells after discarding the outliers. If, as an example, we choose as value of very low probability *ε* = 0.05 then 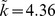 and we will assign all values of cytokines concentration over the threshold 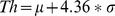 to an Ag-stimulated well because such values would derive from an un-stimulated well only with a low probability. As a consequence, considering the Ag-stimulated wells, when the cytokine concentration is over the exceptionality threshold *Th*, the well will be declared active (*i.e*., Ag-activated).

**Figure 4:**
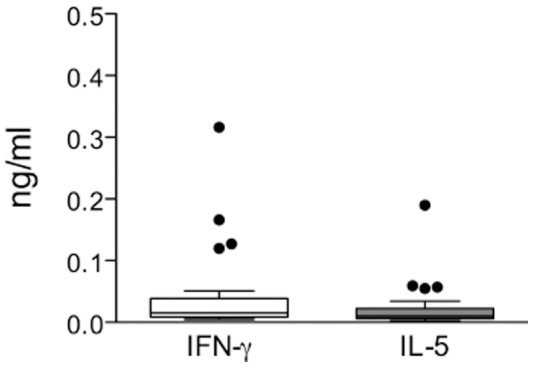
Box and whisker plots of the distribution of basal cytokine production by CD4^+^ T cell cultures. The bottom and top of the box are the 25^th^ (Q1) and 75^th^ percentile (Q3), the lower and upper quartiles, respectively, and the band near the middle of the box is the 50^th^ percentile (the median). The ends of the whiskers represent the smallest datum still within 1.5 the Interquartile Range (IQR = Q3−Q1) of the lower quartile, and the largest datum still within 1.5 IQR of the upper quartile. Outliers are represented as black dots.

Table 1 reports the calculation of independent exceptionality thresholds for IFN-γ and IL-5 release at day 14, based on the estimate of the mean *µ* and the standard deviation *σ* of the distribution of the basal cytokine production in the un-stimulated wells (without outliers). An example of the distribution of IFN-γ and IL-5 basal values of the un-stimulated wells for a representative donor (#12) is also reported in Fig. 4. In this example, the quartile of the distribution of IFN-γ concentration in the un-stimulated wells are Q1 = 9.07, Q2 = 15.65 and Q3 = 37.57. The data over Q3+1.5* (Q3-Q1)(in this example equal to 80.32) are the outliers. The mean µ of the non outliers is 18.66 and the standard deviation *σ* is 13.6, so setting *ε* = 0.05, *κ* is 4.36 and the exceptionality threshold *Th* is 18.66+4.36*13.60 = 77.93. Therefore, the Ag-stimulated wells in which IFN-γ release is greater or equal to 77.93 can be declared active. For IL-5 the quartile of concentration distribution in the un-stimulated wells are Q1 = 6.00, Q2 = 9.50 and Q3 = 21.25. The data over Q3+1.5* (Q3-Q1)(in this example equal to 44.12) are the outliers. The mean *µ* of the non outliers is 10.77 and the standard deviation *σ* is 8.23, so setting *ε* = 0.05, *κ* is 4.36 and the exceptionality threshold *Th* is 10.77+4.36*8.23 = 46.63. Therefore, the Ag-stimulated wells in which IL-5 release is greater or equal to 46.63 can be declared active. The values of the thresholds in the un-stimulated wells for each donor, reported in Table 1, were then used to calculate the number of inactive wells for IFN-γ and IL-5, respectively, in the HA- and EBNA2-stimulated wells (Table S8).

**Table 1:** Mean, standard deviation (after discarding of the outliers) and threshold for IFN-γ and IL-5 concentration at day 14 in the un-stimulated wells.

Donor #	IFN-γ (pg/ml)			IL-5 (pg/ml)		
	Mean	SD	Threshold	Mean	SD	Threshold
11	22.62	6.76	52.10	16.51	10.76	63.40
12	18.66	13.60	77.93	10.77	8.23	46.63
13	8.90	4.02	26.43	n.p.^a^	n.p.	n.p.
14	11.91	5.61	36.36	11.79	4.10	29.68
15	23.04	16.87	96.59	57.00	55.85	300.43
16	2.69	2.22	12.38	57.85	55.55	300.00
17	7.49	5.33	30.72	18.36	22.72	117.11

a n.p., not performed.

### Estimation of the Frequency of Ag-activated Cytokine-producing CD4^+^ T cells

#### Point and interval estimation

After having established for each donor the exceptionality threshold (Table 1), the number of inactive/active in the Ag-stimulated wells (Table S8) and the number of cells per well (reported in Tables S1, S2, S3, S4, S5, S6, S7), to calculate the frequency of Ag-stimulated CD4^+^ T cells we applied the estimation procedure reported in the Materials and Methods: the results are reported in Table 2 and Table 3.

**Table 2:** Point and interval frequencies of Ag-activated cytokine-secreting CD4^+^ T cells in the HA-stimulated wells.

Donor #	IFN-γ			IL-5		
	Lower^a^	Punctual	Upper	Lower	Punctual	Upper
11	39.3	67.1	109.0	1.9	6.1	14.2
12	44.2	76.8	129.0	2.7	7.4	16.2
13	n.p.^b^	n.p.	n.p.	n.p.	n.p.	n.p.
14	8.6	17.0	30.0	4.4	10.3	20.5
15	35.3	59.7	95.8	26.0	44.1	69.9
16	35.3	59.7	95.8	0.3	2.3	8.3
17	41.0	65.4	98.3	15.8	30.1	51.7

a Lower, punctual and upper frequencies are indicated as the number of IFN-γ and IL-5 secreting cells/10^6^ total CD4^+^ T cells.

b n.p., not performed.

**Table 3:** Point and interval frequencies of Ag-activated cytokine-secreting CD4^+^ T cells in the EBNA-stimulated wells.

Donor #	IFN-γ			IL-5		
	Lower^a^	Punctual	Upper	Lower	Punctual	Upper
11	25.9	44.0	69.9	1.3	4.8	12.2
12	31.7	53.6	85.4	1.9	6.1	14.2
13	58.6	113.0	236.0	n.p.^b^	n.p.	n.p.
14	11.1	21.0	35.6	0.7	3.5	10.3
15	19.2	33.4	53.8	6.3	13.5	25.0
16	23.5	40.1	63.8	2.7	7.4	16.2
17	38.3	61.6	93.3	4.6	12.8	27.9

a Lower, punctual and upper frequencies are indicated as the number of IFN-γ and IL-5 secreting cells/10^6^ total CD4 T cells.

b n.p., not performed.

To take into account the inter-experimental variability we decided also to provide interval estimation of the values of the frequency. To this aim we applied the Clopper-Pearson method to define a confidence interval of level (1-α) for the frequency *f*. The confidence interval length decreases as the number *N* of seeded cells augments and with increasing numbers of inactive wells (*y_0_*). For our purposes, a 95% confidence interval was chosen, corresponding to *α* = 0.05.

**Figure 5:**
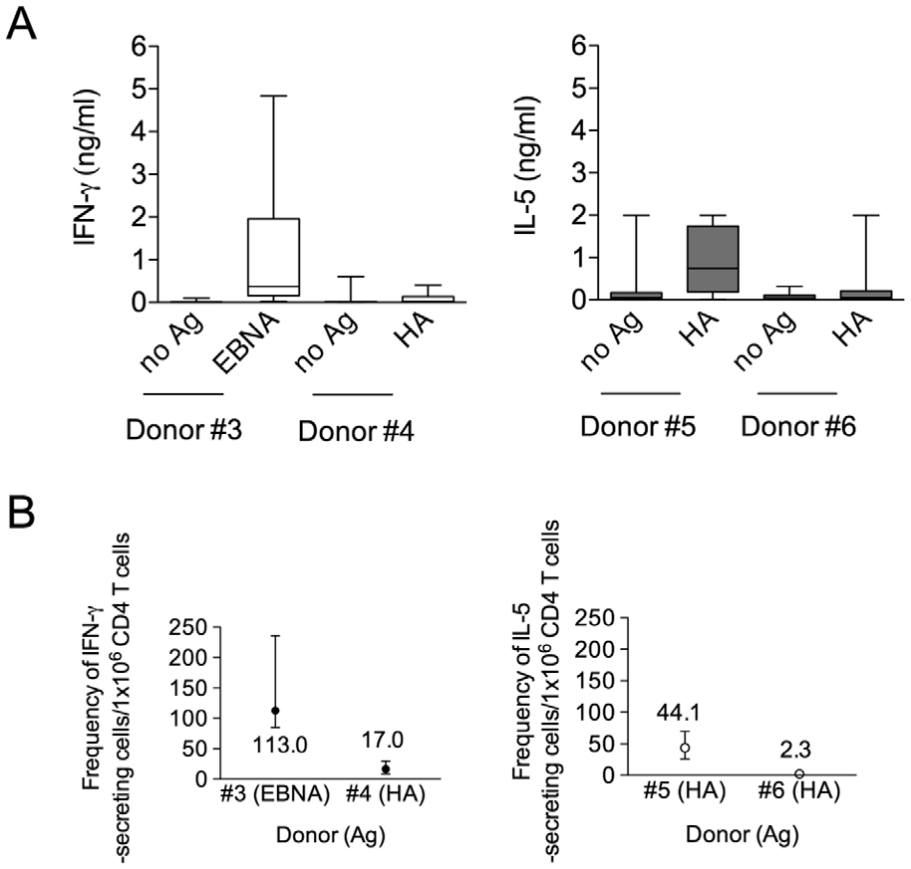
Point and interval estimations of Ag-specific CD4^+^ T cells frequencies. (A) Box and whisker plots of the distribution of cytokines (IFN-γ and IL-5) release by CD4^+^ T cell cultures within the un-stimulated and Ag-stimulated wells of representative donors. (B) Representation of point and interval frequencies of Ag-specific CD4^+^ T cells for representative donors.

Figure 5 reports box and whisker plots of cytokines distribution in the un-stimulated and in the Ag-stimulated wells for both IFN-γ (Fig. 5A, left) and IL-5 (Fig. 5A, right) in donors representative of higher and lower frequency of viral-specific CD4^+^ T cells (Fig. 5B). The estimates of interval frequencies for all donors were calculated and are reported along with the punctual ones in Table 2 and Table 3.

Finally, we converted the proposed statistical method into a ready-to-use Excel worksheet (Table S9), which automatically calculate the exceptionality threshold, the point and interval frequencies, once *N* (*i.e.*, the number of seeded cells/well), *ε* (*i.e*., the probability defining the exceptionality threshold) and *α* (*i.e*., the confidence level of interval estimates) are set and the experimental data from the wells are input in the columns of un-stimulated and Ag-stimulated wells. The worksheet is designed for a maximum of 60 wells for the un-stimulated as well as for the Ag-stimulated wells and it automatically infers the number *n* of wells seeded.

## Discussion

We report a culture method combined with a statistical analysis strategy that allows the estimate of low frequency Ag-specific memory CD4^+^ T cells. Indeed, frequency of IFN-γ secreting HA- and EBNA2- specific CD4^+^ T cells as low as 0.0017% and 0.0021% of total CD4^+^ T cells, respectively, and frequency of IL-5 secreting HA- and EBNA2- specific CD4^+^ T cells as low as 0.00023% and 0.00035% of total CD4^+^ T cells, respectively, could be detected in a cohort of healthy donors.

Important issues to be considered in the evaluation of the power of a detection system are the specificity and the sensitivity. Concerning the specificity, in our experimental protocol 0.9×10^6^ CD4^+^ T cells are cultured in 30 independent wells (30×10^4^/well) with peptide-pulsed APCs and an equal number of CD4^+^ T cells, distributed in the same number of wells, are cultured in the absence of the peptide (*i.e.*, un-pulsed APCs). The specificity derives from the comparison between the two groups (+Ag and −Ag), once criteria to define the background level cytokine secretion in the −Ag group have been established. Since spontaneous cytokine release of CD4^+^ T cells in the presence of un-pulsed APCs is heterogeneous and highly varies among different subjects, the background level of cytokine release needs to be tailored. In our system the background level of cytokine secretion is established for each donor by the calculation of a proper exceptionality threshold beyond which a value of cytokine release would belong to the distribution of the un-stimulated wells with a very low probability. As a consequence, all the values within the stimulated-wells that are over this threshold (*i.e.*, are exceptional) are considered specifically activated. Therefore, the introduction of the exceptionality threshold in our statistical analysis to estimate the number of activated wells is an important innovation because it takes into account the patient-specific distribution of cytokines release in the un-stimulated wells.

Concerning the sensitivity, the theoretical detection limits of our procedure depends on the total number of CD4^+^ T cells studied. The condition of (*n−1*) inactive wells in the stimulated-wells represents the lower detection limit, because the corresponding frequency (*f* = 1.13×10^−6^ for 29 inactive wells out of 30) is the lowest point estimation calculable above 0. On the other hand, the condition of 1 inactive well in the Ag-stimulated wells represents the upper detection limit, because the corresponding frequency (*f* = 1.13×10^−4^ for 1 inactive well out of 30) is the highest point estimation. In the opposite cases of *n* inactive wells or 0 inactive wells, point estimates of frequencies are trivial, but we can compute 95% confidence interval estimates, which have quite broad limits. By doubling the total number of cells tested (60 wells of 30×10^4^ CD4^+^ T cells/well), the lower and upper detection limits become *f* = 5.60×10^−7^ and *f* = 1.36×10^−4^, respectively. In both formats (30 and 60 wells), frequency estimate covers at least a two-log range.

Beside the calculation of the exceptionality threshold, another important statistical tool in our system is the calculation of both the punctual and interval frequencies. Indeed, the calculation the interval frequencies with the lower and the upper limits reflects the possible range of inter-experimental variability.

Previous studies have evaluated the frequency of HA_306–318_-specific CD4^+^ T cells of healthy normal individuals by the use HLA class II multimers both directly *ex-vivo* staining and after one week *in vitro* re-stimulation. One study [27] reported estimates of DRB1*0401 tetramer-specific T cells frequencies ranging between 30 and 50×10^−6^. Another study [28], found frequencies ranging from 1.2 to 60×10^−6^ using DRB1*0101 tetramers. A third study [29] also reported similar frequencies using both DRB1*0401 and DRB1*0101 tetramers. In the present study, we used the same promiscuous HA peptide in an HLA-DR unselected population (*i.e*., subjects bearing disparate HLA-DR alleles) of healthy donors and found punctual estimate of IFN-γ secreting CD4^+^ T cells ranging from 17 and 77×10^−6^, which are values very much compatible with the ones reported in the other studies. Paired values, ranging from 2.3 and 44.1×10^−6^, were found for IL-5 secreting cells. In three donors (#11, #12, and #16) the frequency of IFN-γ secreting cells highly exceeded the IL-5 secreting ones. In other three donors (#14, #15 and #17) the frequency of IL-5 secreting cells were instead comparable or about half of the IFN-γ secreting cells, suggesting the expansion in different donors of Th1 and Th0 or Th1/Th2 subsets. For the EBNA2-specific CD4^+^ T cells we found frequencies of IFN-γ secreting cells ranging from 21 to 113×10^−6^ and from 3.5 to 13.5×10^−6^ for IL-5. When we considered paired IFN-γ/IL-5 responses in the case of EBNA2 specific CD4^+^ T cells the frequency of IL-5 secreting cells was usually much lower compared to IFN-γ, suggesting that the response to EBV in these subjects was more polarized toward a Th1 type. Although, to our knowledge EBNA2-specific CD4^+^ T cell frequencies have been not reported so far, a study [30], which evaluated the response against the EBNA1 protein also found primarily Th1 type responses with frequencies that were in the order of few hundreds per million cells but in this case responses to multiple epitopes were studied.

With the advent of Ag and peptide specific vaccination therapies, a need for immune-monitoring methods to be applied in large scale clinical trials has emerged as a fundamental prerequisite for correlative studies to compare induction/expansion of Ag-specific immune responses and clinical efficacy [31]. Methods that are both quantitative and qualitative should be employed. The most widely used assay is the IFN-γ ELISPOT and, recently, panels of researcher from different centers have began important useful comparisons among laboratories in order to harmonize the experimental protocols and read outs from different reading instruments [32].

We believe that, although the method we describe has of course some cons (such as the inability to detect non-proliferating/non-expanding cells and the *ex-vivo* phenotype) there are several pros that may indicate its application in large scale clinical trials that employs as immunogens protein Ags or peptides corresponding to promiscuous epitopes. Indeed, given that the frequencies of Ag-specific memory CD4^+^ T cells are higher than those of Ag-specific naïve CD4^+^ T cells (the latter is in the order of few to 70 cells per million CD4^+^ naïve T cells [25], [26]) and provided that our system can only detect recall responses, we believe that 1×10^6^ total CD4^+^ T cells is a reasonable number to study most memory frequencies. This allows the use of small PBMCs samples, usually less than 10×10^6^. The method is easy to perform and not labor-intensive, it does not require dedicated facilities or complicated reading instrumentations neither costly material. Results are obtained in two weeks and multiple functional assays (*i.e.*, cytokine arrays and proliferation assays) can be performed. Moreover, since cells growing in the Ag-activated wells most likely derive from the expansion of one precursor; in the case in which proliferation assays are not performed, growing cells can be further cultured and easily cloned for additional functional studies.

Collectively, we describe here a method for monitoring of Ag-specific CD4^+^ T cell responses that would be of advantage in large scale clinical trials of vaccination with protein or peptide Ags. Its use is greatly facilitated by the enclosed ready-to-use Excel application/worksheet with integrated the statistical analysis developed in this study.

## Supporting Information

Tables S1 Values of single wells cytokines (IFN-γ and IL-5) production measured by ELISA in un-stimulated or HA- or EBNA-stimulated wells for donors #11, #12, #13, #14, #15, #16 and #17, respectively. Values are the mean of duplicates.
(DOC)

Table S2 Values of single wells cytokines (IFN-γ and IL-5) production measured by ELISA in un-stimulated or HA- or EBNA-stimulated wells for donors #11, #12, #13, #14, #15, #16 and #17, respectively. Values are the mean of duplicates.
(DOC)

Table S3 Values of single wells cytokines (IFN-γ and IL-5) production measured by ELISA in un-stimulated or HA- or EBNA-stimulated wells for donors #11, #12, #13, #14, #15, #16 and #17, respectively. Values are the mean of duplicates.
(DOC)

Table S4 Values of single wells cytokines (IFN-γ and IL-5) production measured by ELISA in un-stimulated or HA- or EBNA-stimulated wells for donors #11, #12, #13, #14, #15, #16 and #17, respectively. Values are the mean of duplicates.
(DOC)

Table S5 Values of single wells cytokines (IFN-γ and IL-5) production measured by ELISA in un-stimulated or HA- or EBNA-stimulated wells for donors #11, #12, #13, #14, #15, #16 and #17, respectively. Values are the mean of duplicates.
(DOC)

Table S6 Values of single wells cytokines (IFN-γ and IL-5) production measured by ELISA in un-stimulated or HA- or EBNA-stimulated wells for donors #11, #12, #13, #14, #15, #16 and #17, respectively. Values are the mean of duplicates.
(DOC)

Table S7 Values of single wells cytokines (IFN-γ and IL-5) production measured by ELISA in un-stimulated or HA- or EBNA-stimulated wells for donors #11, #12, #13, #14, #15, #16 and #17, respectively. Values are the mean of duplicates.
(DOC)

Table S8 Calculation of inactive wells for IFN-γ and IL-5 secretion at day 14 cultures in the Ag-stimulated wells for all donors (#11, #12, #13, #14, #15, #16, #17).
(DOC)

Table S9 Excel application and instructions for the calculation of Ag-specific CD4^+^ T cell frequency.
(XLS)
