# Supplementary material for: Estimating Point and Interval Frequency of Antigen-Specific CD4+ T Cells Based on Short In Vitro Expansion and Improved Poisson Distribution Analysis
Source: PLoS One. 2012 Aug 7;7(8):e42340. doi: 10.1371/journal.pone.0042340 (PMC3413706; doi:10.1371/journal.pone.0042340)
Supplement: Tables S1 — Values of single wells cytokines (IFN-γ and IL-5) production measured by ELISA in un-stimulated or HA- or EBNA-stimulated wells for donors #11, #12, #13, #14, #15, #16 and #17, respectively. Values are the mean of duplicates. (DOC) [file pone.0042340.s001.doc]

**Table S1**. Single well cytokines release was measured by ELISA. Values are the mean of duplicates.

| Donor #11 | | |  | 30,000 CD4^+^ T cells/well | | | | 30 wells/condition | | |
| --- | --- | --- | --- | --- | --- | --- | --- | --- | --- | --- |
| IFN-γ | (pg/ml) |  |  |  |  | IL-5 | (pg/ml) |  |  |  |
| n.s.^a^ |  |  |  |  |  | n.s. |  |  |  |  |
| 2002.00 | 238.30 | 29.09 | 20.91 | 22.55 |  | 45.39 | 625.38 | 276.50 | 9.91 | 17.18 |
| 19.64 | 23.64 | 24.55 | 29.27 | 19.45 |  | 10.84 | 130.95 | 12.16 | 6.74 | 12.82 |
| 28.18 | 27.27 | 34.03 | 1915.23 | 12.73 |  | 17.58 | 14.14 | 13.22 | 513.60 | 10.57 |
| 20.00 | 16.73 | 68.53 | 10.73 | 193.21 |  | 9.25 | 23.52 | 32.13 | 10.31 | 290.83 |
| 33.16 | 19.27 | 13.27 | 14.18 | 24.73 |  | 12.69 | 15.33 | 488.50 | 7.67 | 12.29 |
| 36.71 | 23.09 | 21.82 | 16.73 | 23.82 |  | 47.83 | 12.56 | 19.30 | 8.46 | 14.27 |
| HA |  |  |  |  |  | HA |  |  |  |  |
| 1725.13 | 1840.97 | 1630.89 | 71.18 | 1691.75 |  | 15.63 | 9.08 | 7.82 | 9.43 | 14.02 |
| 2002.00 | 1918.54 | 823.83 | 981.20 | 229.24 |  | 10.69 | 134.29 | 18.05 | 10.23 | 16.09 |
| 1738.22 | 38.93 | 63.24 | 1357.98 | 1155.10 |  | 11.15 | 10.23 | 19.08 | 9.20 | 18.39 |
| 103.21 | 2002.00 | 1253.27 | 19.09 | 34.22 |  | 8.85 | 8.51 | 4.48 | 5,98 | 26,21 |
| 1980.37 | 604.36 | 1751.31 | 1415.58 | 772.15 |  | 28,28 | 38,32 | 389,65 | 87.43 | 55.94 |
| 2002.00 | 49.60 | 306.98 | 590.61 | 1195.68 |  | 75.50 | 23.91 | 20.34 | 973.96 | 11.72 |
| EBNA |  |  |  |  |  | EBNA |  |  |  |  |
| 1359.08 | 317.60 | 28.19 | 39.64 | 383.24 |  | 17.27 | 11.14 | 17.27 | 14.82 | 14.82 |
| 476.19 | 21.53 | 25.06 | 312.85 | 188.88 |  | 29.30 | 1417.97 | 97.86 | 7.96 | 9.18 |
| 132.37 | 1649.50 | 163.98 | 115.55 | 27.35 |  | 21.06 | 14.33 | 9.31 | 51.70 | 94.57 |
| 657.89 | 35.33 | 121.79 | 369.83 | 2002.00 |  | 16.04 | 17.14 | 27.31 | 62.29 | 1500.98 |
| 1333.33 | 1840.49 | 189.51 | 153.93 | 70.08 |  | 11.27 | 9.06 | 12.12 | 9.67 | 11.27 |
| 1870.53 | 46.21 | 1484.98 | 25.59 | 145.35 |  | 12.98 | 12.98 | 35.70 | 12.24 | 15.55 |

^a^n.s., not stimulated (un-stimulated)
