# Supplementary material for: Estimating Point and Interval Frequency of Antigen-Specific CD4+ T Cells Based on Short In Vitro Expansion and Improved Poisson Distribution Analysis
Source: PLoS One. 2012 Aug 7;7(8):e42340. doi: 10.1371/journal.pone.0042340 (PMC3413706; doi:10.1371/journal.pone.0042340)
Supplement: Table S2 — Values of single wells cytokines (IFN-γ and IL-5) production measured by ELISA in un-stimulated or HA- or EBNA-stimulated wells for donors #11, #12, #13, #14, #15, #16 and #17, respectively. Values are the mean of duplicates. (DOC) [file pone.0042340.s002.doc]

**Table S2**. Single well cytokines release was measured by ELISA. Values are the mean of duplicates.

| Donor #12 | | |  | 30,000 CD4^+^ T cells/well | | | | 30 wells/condition | | |
| --- | --- | --- | --- | --- | --- | --- | --- | --- | --- | --- |
| IFN-γ | (pg/ml) |  |  |  |  | IL-5 | (pg/ml) |  |  |  |
| ^a^n.s. |  |  |  |  |  | n.s. |  |  |  |  |
| 315.59 | 17.09 | 6.37 | 17.62 | 48.20 |  | 21.83 | 11.83 | 26.15 | 58.63 | 33.82 |
| 38.07 | 5.06 | 14.22 | 4.80 | 120.16 |  | 14.95 | 19.25 | 189.55 | 5.91 | 14.52 |
| 18.49 | 9.33 | 8.98 | 5.84 | 40.22 |  | 14.52 | 10.32 | 5.05 | 3.44 | 5.70 |
| 14.04 | 8.11 | 19.19 | 32.33 | 166.37 |  | 7.85 | 3.01 | 5.91 | 6.34 | 6.02 |
| 14.13 | 8.46 | 36.01 | 7.06 | 22.15 |  | 24.09 | 4.09 | 4.84 | 1.94 | 57.06 |
| 50.62 | 126.91 | 12.82 | 14.13 | 11.95 |  | 6.34 | 54.93 | 3.33 | 9.14 | 9.89 |
| HA |  |  |  |  |  | HA |  |  |  |  |
| 297.15 | 136.64 | 389.23 | 347.57 | 696.08 |  | 31.54 | 13.15 | 21.41 | 19.02 | 25.33 |
| 42.15 | 107.56 | 440.09 | 1527.75 | 1191.39 |  | 12.07 | 11.63 | 45.58 | 17.61 | 10.87 |
| 196.02 | 123.77 | 177.74 | 101.71 | 1338.62 |  | 47.13 | 61.52 | 15.43 | 16.63 | 31.57 |
| 442.62 | 764.71 | 202.03 | 1175.54 | 158.72 |  | 50.82 | 20.65 | 25.00 | 1205.99 | 25.87 |
| 62.91 | 69.19 | 537.99 | 280.55 | 1050.50 |  | 11.85 | 9.57 | 31.22 | 10.65 | 98.25 |
| 1337.49 | 244.94 | 379.00 | 231.46 | 158.72 |  | 54.39 | 43.68 | 11.52 | 35.83 | 26.98 |
| EBNA |  |  |  |  |  | EBNA |  |  |  |  |
| 66.39 | 9.63 | 8.26 | 458.66 | 831.10 |  | 68.09 | 18.00 | 10.47 | 211.17 | 75.57 |
| 85.63 | 39.83 | 945.28 | 180.43 | 173.10 |  | 10.04 | 4.47 | 12.44 | 2.95 | 11.67 |
| 102.73 | 218.06 | 11.28 | 367.68 | 842.13 |  | 10.80 | 37.62 | 11.24 | 14.73 | 12.98 |
| 215.69 | 924.80 | 428.03 | 145.53 | 756.30 |  | 6.76 | 10.15 | 5.02 | 5.56 | 50.40 |
| 467.86 | 913.78 | 1361.66 | 119.16 | 391.52 |  | 39.02 | 4.04 | 357.25 | 18.76 | 20.40 |
| 495.02 | 267.95 | 63.91 | 225.27 | 1016.34 |  | 7.31 | 31.93 | 36.77 | 10.15 | 39.02 |

^a^n.s., not stimulated (un-stimulated)
