# Supplementary material for: Estimating Point and Interval Frequency of Antigen-Specific CD4+ T Cells Based on Short In Vitro Expansion and Improved Poisson Distribution Analysis
Source: PLoS One. 2012 Aug 7;7(8):e42340. doi: 10.1371/journal.pone.0042340 (PMC3413706; doi:10.1371/journal.pone.0042340)
Supplement: Table S3 — Values of single wells cytokines (IFN-γ and IL-5) production measured by ELISA in un-stimulated or HA- or EBNA-stimulated wells for donors #11, #12, #13, #14, #15, #16 and #17, respectively. Values are the mean of duplicates. (DOC) [file pone.0042340.s003.doc]

**Table S3**. Single well cytokines release was measured by ELISA. Values are the mean of duplicates.

| Donor #13 | | |  | 30,000 CD4^+^ T cells/well | | | | 30 wells/condition | | |
| --- | --- | --- | --- | --- | --- | --- | --- | --- | --- | --- |
| IFN-γ | (pg/ml) |  |  |  |  | IL-5 | (pg/ml) |  |  |  |
| n.s.^a^ |  |  |  |  |  | n.s. |  |  |  |  |
| 17.11 | 14.88 | 10.41 | 7.44 | 6.69 |  | 45.33 | 195.50 | 76.57 | 61.28 | 74.29 |
| 15.62 | 10.79 | 5.21 | 5.95 | 5.58 |  | 47.58 | 45.33 | 0.00 | 27.58 | 24.33 |
| 27.52 | 24.92 | 11.90 | 10.04 | 7.81 |  | 168.21 | 78.19 | 48.00 | 51.33 | 40.67 |
| 11.90 | 8.18 | 3.72 | 9.30 | 93.78 |  | 45.42 | 186.14 | 25.67 | 33.96 | 60.27 |
| 5.21 | 2.98 | 2.23 | 4.83 | 8.93 |  | 38.67 | 7.50 | 20.75 | 1.88 | 31.00 |
| 9.67 | 8.93 | 9.30 | 8.55 | 17.11 |  | 50.67 | 42.67 | 32.67 | 11.25 | 77.50 |
| EBNA |  |  |  |  |  | EBNA |  |  |  |  |
| 60.62 | 2903.61 | 2010.84 | 2257.83 | 158.09 |  | 34.11 | 97.27 | 95.82 | 223.80 | 349.85 |
| 84.42 | 655.35 | 301.36 | 41.65 | 845.87 |  | 304.97 | 25.97 | 392.41 | 30.73 | 12.63 |
| 4832.35 | 1537.71 | 1239.68 | 1940.21 | 380.05 |  | 172.65 | 160.29 | 30.77 | 138.65 | 154.19 |
| 4282.05 | 355.85 | 2362.65 | 1183.25 | 1476.33 |  | 239.32 | 28.36 | 159.66 | 100.59 | 142.71 |
| 363.72 | 165.28 | 215.98 | 156.40 | 24.55 |  | 289.75 | 3.16 | 68.86 | 3.95 | 6.32 |
| 251.05 | 74.38 | 2521.69 | 208.92 | 109.45 |  | 18.95 | 223.92 | 18.16 | 5.53 | 139.68 |

^a^n.s., not stimulated (un-stimulated)
