# Supplementary material for: Estimating Point and Interval Frequency of Antigen-Specific CD4+ T Cells Based on Short In Vitro Expansion and Improved Poisson Distribution Analysis
Source: PLoS One. 2012 Aug 7;7(8):e42340. doi: 10.1371/journal.pone.0042340 (PMC3413706; doi:10.1371/journal.pone.0042340)
Supplement: Table S4 — Values of single wells cytokines (IFN-γ and IL-5) production measured by ELISA in un-stimulated or HA- or EBNA-stimulated wells for donors #11, #12, #13, #14, #15, #16 and #17, respectively. Values are the mean of duplicates. (DOC) [file pone.0042340.s004.doc]

**Table S4**. Single well cytokines release was measured by ELISA. Values are the mean of duplicates.

| Donor #14 | | |  | 30,000 CD4^+^ T cells/well | | | | 30 wells/condition | | |
| --- | --- | --- | --- | --- | --- | --- | --- | --- | --- | --- |
| IFN-γ | (pg/ml) |  |  |  |  | IL-5 | (pg/ml) |  |  |  |
| n.s.^a^ |  |  |  |  |  | n.s. |  |  |  |  |
| 12.58 | 16.97 | 38.66 | 15.45 | 15.76 |  | 14.32 | 19.51 | 11.73 | 7.89 | 11.39 |
| 122.67 | 8.18 | 11.06 | 601.31 | 11.52 |  | 12.29 | 8.23 | 6.99 | 7.78 | 11.73 |
| 13.48 | 10.61 | 5.00 | 7.58 | 7.12 |  | 14.89 | 63.08 | 9.47 | 16.02 | 8.80 |
| 27.88 | 6.67 | 3.33 | 4.24 | 13.94 |  | 17.26 | 6.43 | 5.41 | 11.17 | 19.40 |
| 10.45 | 44.67 | 13.18 | 6.21 | 12.42 |  | 11.39 | 9.59 | 10.38 | 8.57 | 11.17 |
| 16.67 | 22.58 | 13.18 | 11.67 | 217.73 |  | 13.98 | 17.48 | 9.14 | 9.25 | 20.19 |
| HA |  |  |  |  |  | HA |  |  |  |  |
| 134.93 | 12.10 | 14.75 | 19.23 | 406.17 |  | 42.58 | 76.10 | 18.84 | 22.44 | 162.90 |
| 12.60 | 12.43 | 319.72 | 13.26 | 28.67 |  | 16.44 | 11.16 | 160.93 | 8.64 | 10.20 |
| 20.55 | 78.14 | 9.45 | 8.62 | 11.44 |  | 13.20 | 13.80 | 13.44 | 10.68 | 10.92 |
| 314.51 | 223.24 | 17.40 | 16.41 | 199.60 |  | 9.24 | 20.52 | 9.24 | 16.80 | 16.56 |
| 12.10 | 146.97 | 33.17 | 17.07 | 303.00 |  | 12.60 | 9.72 | 48.17 | 39.36 | 31.32 |
| 36.43 | 33.53 | 40.43 | 54.70 | 17.90 |  | 16.32 | 81.96 | 15.24 | 15.36 | 16.80 |
| EBNA |  |  |  |  |  | EBNA |  |  |  |  |
| 18.93 | 331.72 | 93.44 | 31.02 | 1997.84 |  | 9.51 | 7.20 | 18.17 | 9.15 | 7.93 |
| 1196.68 | 126.13 | 76.13 | 61.99 | 2002.00 |  | 7.80 | 33.53 | 18.05 | 2.80 | 6.83 |
| 79.84 | 11.36 | 8.45 | 7.28 | 11.36 |  | 167.77 | 10.24 | 10.61 | 11.34 | 8.05 |
| 26.36 | 327.84 | 15.87 | 18.64 | 13.69 |  | 6.34 | 11.46 | 3.66 | 3.41 | 6.34 |
| 175.87 | 10.63 | 6.84 | 9.17 | 71.36 |  | 26.71 | 9.88 | 17.93 | 5.73 | 6.34 |
| 19.37 | 11.65 | 97.15 | 44.00 | 19.95 |  | 30.61 | 5.98 | 10.12 | 9.76 | 13.78 |

^a^n.s., not stimulated (un-stimulated)
