# Supplementary material for: Estimating Point and Interval Frequency of Antigen-Specific CD4+ T Cells Based on Short In Vitro Expansion and Improved Poisson Distribution Analysis
Source: PLoS One. 2012 Aug 7;7(8):e42340. doi: 10.1371/journal.pone.0042340 (PMC3413706; doi:10.1371/journal.pone.0042340)
Supplement: Table S5 — Values of single wells cytokines (IFN-γ and IL-5) production measured by ELISA in un-stimulated or HA- or EBNA-stimulated wells for donors #11, #12, #13, #14, #15, #16 and #17, respectively. Values are the mean of duplicates. (DOC) [file pone.0042340.s005.doc]

**Table S5**. Single well cytokines release was measured by ELISA. Values are the mean of duplicates.

| Donor #15 | | |  | 30,000 CD4^+^ T cells/well | | | | 30 wells/condition | | |
| --- | --- | --- | --- | --- | --- | --- | --- | --- | --- | --- |
| IFN-γ | (pg/ml) |  |  |  |  | IL-5 | (pg/ml) |  |  |  |
| n.s.^a^ |  |  |  |  |  | n.s. |  |  |  |  |
| 19.22 | 9.72 | 8.54 | 9.07 | 37.31 |  | 52.40 | 86.00 | 12.37 | 7.37 | 68.00 |
| 23.27 | 11.00 | 15.16 | 45.66 | 29.06 |  | 34.80 | 13.16 | 2002.00 | 1100.99 | 167.46 |
| 19.22 | 10.68 | 23.59 | 1655.29 | 119.88 |  | 36.13 | 34.53 | 29.49 | 436.62 | 54.80 |
| 37.09 | 8.43 | 6.19 | 23.70 | 24.45 |  | 36.43 | 27.89 | 11.58 | 239.04 | 44.93 |
| 71.14 | 5.98 | 3.10 | 411.33 | 29.58 |  | 37.20 | 42.53 | 3.16 | 167.46 | 1849.39 |
| 1658.70 | 62.95 | 26.16 | 15.59 | 23.49 |  | 2002.00 | 56.67 | 77.20 | 35.33 | 51.07 |
| HA |  |  |  |  |  | HA |  |  |  |  |
| 68.54 | 576.30 | 2002.00 | 8.01 | 1981.03 |  | 156.42 | 2002.00 | 731.22 | 191.31 | 1607.35 |
| 21.99 | 796.68 | 1400.40 | 1627.91 | 77.61 |  | 7.80 | 1444.57 | 2002.00 | 727.70 | 18.30 |
| 448.62 | 809.48 | 240.95 | 1219.92 | 331.08 |  | 758.22 | 335.67 | 2002.00 | 957.63 | 160.12 |
| 1551.06 | 480.26 | 968.25 | 1863.50 | 1956.52 |  | 1027.42 | 2002.00 | 578.05 | 396.54 | 92.54 |
| 24.12 | 1604.15 | 231.71 | 1705.76 | 307.96 |  | 10.80 | 2002.00 | 592.14 | 1710.04 | 1176.20 |
| 987.98 | 482.14 | 1752.28 | 114.45 | 1716.89 |  | 957.75 | 67.26 | 368.91 | 2002.00 | 1822.64 |
| EBNA |  |  |  |  |  | EBNA |  |  |  |  |
| 37.50 | 446.92 | 1266.72 | 2002.00 | 342.89 |  | 37.78 | 369.91 | 179.54 | 18.75 | 1142.18 |
| 116.83 | 765.07 | 37.96 | 900.31 | 1923.36 |  | 241.05 | 2002.00 | 3.00 | 169.48 | 0.00 |
| 258.23 | 77.89 | 205.58 | 1581.72 | 40.41 |  | 58.41 | 24.00 | 1375.08 | 85.94 | 2002.00 |
| 9.96 | 1884.84 | 2002.00 | 52.05 | 66.22 |  | 9.00 | 1924.16 | 2002.00 | 175.07 | 369.12 |
| 903.40 | 846.21 | 190.05 | 1221.40 | 9.32 |  | 161.65 | 52.73 | 38.67 | 2002.00 | 40.77 |
| 15.96 | 71.14 | 585.39 | 15.70 | 341.83 |  | 219.43 | 808.69 | 111.11 | 8.75 | 10.50 |

^a^n.s., not stimulated (un-stimulated)
