# Supplementary material for: Estimating Point and Interval Frequency of Antigen-Specific CD4+ T Cells Based on Short In Vitro Expansion and Improved Poisson Distribution Analysis
Source: PLoS One. 2012 Aug 7;7(8):e42340. doi: 10.1371/journal.pone.0042340 (PMC3413706; doi:10.1371/journal.pone.0042340)
Supplement: Table S6 — Values of single wells cytokines (IFN-γ and IL-5) production measured by ELISA in un-stimulated or HA- or EBNA-stimulated wells for donors #11, #12, #13, #14, #15, #16 and #17, respectively. Values are the mean of duplicates. (DOC) [file pone.0042340.s006.doc]

**Table S6**. Single well cytokines release was measured by ELISA. Values are the mean of duplicates.

| Donor #16 | | |  | 30,000 CD4^+^ T cells/well | | | | 30 wells/condition | | |
| --- | --- | --- | --- | --- | --- | --- | --- | --- | --- | --- |
| IFN-γ | (pg/ml) |  |  |  |  | IL-5 | (pg/ml) |  |  |  |
| n.s.^a^ |  |  |  |  |  | n.s. |  |  |  |  |
| 7.65 | 4.80 | 2.96 | 13.47 | 13.47 |  | 196.21 | 24.45 | 16.64 | 40.57 | 15.13 |
| 2.45 | 5.31 | 0.92 | 1.73 | 5.00 |  | 11.60 | 38.45 | 276.57 | 33.23 | 106.50 |
| 7.55 | 5.00 | 2.24 | 1.33 | 2.65 |  | 129.49 | 24.45 | 19.41 | 80.26 | 314.75 |
| 2.35 | 1.73 | 20.51 | 0.92 | 2.86 |  | 82.54 | 12.35 | 116.77 | 248.27 | 17.90 |
| 1.22 | 1.22 | 0.00 | 0.15 | 2.04 |  | 30.98 | 18.40 | 100.80 | 19.16 | 107.64 |
| 1.12 | 17.96 | 1.22 | 0.82 | 5.00 |  | 10.08 | 41.77 | 206.89 | 20.92 | 40.26 |
| HA |  |  |  |  |  | HA |  |  |  |  |
| 1613.49 | 281.82 | 414.50 | 383.55 | 44.02 |  | 242.19 | 253.29 | 50.00 | 50.29 | 11.86 |
| 25.85 | 126.82 | 250.24 | 122.99 | 14.52 |  | 523.58 | 34.62 | 279.90 | 21.86 | 69.61 |
| 109.42 | 43.68 | 50.01 | 11.47 | 16.45 |  | 67.51 | 26.51 | 30.66 | 11.86 | 61.42 |
| 58.16 | 26.68 | 10.78 | 24.33 | 10.51 |  | 133.69 | 210.23 | 8.14 | 15.58 | 166.78 |
| 103.76 | 387.88 | 692.51 | 37.58 | 31.91 |  | 25.12 | 27.67 | 2002.00 | 50.00 | 46.80 |
| 53.41 | 11.89 | 6.50 | 74.17 | 96.26 |  | 227.94 | 26.98 | 219.25 | 12.09 | 34.86 |
| EBNA |  |  |  |  |  | EBNA |  |  |  |  |
| 2002.00 | 553.92 | 2002.00 | 2002.00 | 2002.00 |  | 28.66 | 10.45 | 2002.00 | 225.21 | 2002.00 |
| 2.25 | 1.69 | 0.67 | 0.67 | 8.54 |  | 273.60 | 2002.00 | 47.52 | 72.61 | 25.78 |
| 30.44 | 9.55 | 3.26 | 2002.00 | 1196.61 |  | 118.62 | 148.45 | 26.69 | 111.62 | 26.15 |
| 19.21 | 2002.00 | 2002.00 | 2002.00 | 201.02 |  | 96.09 | 79.99 | 68.17 | 827.23 | 15.54 |
| 2002.00 | 381.46 | 2002.00 | 1987.98 | 2002.00 |  | 623.27 | 60.13 | 37.07 | 85.21 | 79.63 |
| 7.87 | 0.00 | 2002.00 | 198.21 | 1360.68 |  | 220.79 | 41.79 | 2002.00 | 140.30 | 42.97 |

^a^n.s., not stimulated (un-stimulated)
