# Supplementary material for: Estimating Point and Interval Frequency of Antigen-Specific CD4+ T Cells Based on Short In Vitro Expansion and Improved Poisson Distribution Analysis
Source: PLoS One. 2012 Aug 7;7(8):e42340. doi: 10.1371/journal.pone.0042340 (PMC3413706; doi:10.1371/journal.pone.0042340)
Supplement: Table S7 — Values of single wells cytokines (IFN-γ and IL-5) production measured by ELISA in un-stimulated or HA- or EBNA-stimulated wells for donors #11, #12, #13, #14, #15, #16 and #17, respectively. Values are the mean of duplicates. (DOC) [file pone.0042340.s007.doc]

**Table S7**. Single well cytokines release was measured by ELISA. Values are the mean of duplicates.

| Donor #17 | | |  | 10,000 CD4^+^ T cells/well | | | 50 wells/condition | | |
| --- | --- | --- | --- | --- | --- | --- | --- | --- | --- |
| IFN-γ | (pg/ml) |  |  |  |  |  |  |  |  |
| n.s.^a^ |  |  |  |  |  |  |  |  |  |
| 5.77 | 4.12 | 7.25 | 5.11 | 1.98 | 10.43 | 2.14 | 1.86 | 1.36 | 4.00 |
| 7.75 | 21.59 | 15.00 | 23.24 | 12.20 | 11.57 | 8.14 | 7.86 | 4.14 | 10.57 |
| 16.15 | 5.77 | 2.31 | 4.62 | 3.63 | 104.92 | 5.14 | 2.43 | 1.86 | 272.47 |
| 34.86 | 64.05 | 2.16 | 3.40 | 10.05 | 672.92 | 29.94 | 7.46 | 3.30 | 4.31 |
| 7.42 | 6.49 | 11.91 | 5.88 | 10.52 | 48.55 | 5.31 | 10.77 | 17.66 | 8.61 |
| HA |  |  |  |  |  |  |  |  |  |
| 10.05 | 9.74 | 91.20 | 8.97 | 13.76 | 22.39 | 21.39 | 1271.88 | 21.39 | 6.17 |
| 9.43 | 23.20 | 16.39 | 3.25 | 1707.47 | 50.35 | 11.20 | 8.61 | 7.75 | 2002.00 |
| 15.46 | 547.04 | 36.60 | 1905.93 | 274.63 | 158.86 | 30.39 | 74.01 | 28.57 | 409.13 |
| 275.31 | 11.60 | 4.02 | 12.37 | 114.60 | 2002.00 | 303.04 | 46.90 | 1906.74 | 57.90 |
| 4.02 | 6.96 | 8.51 | 196.10 | 283.31 | 108.46 | 16.08 | 1970.59 | 79.49 | 24.83 |
| EBNA |  |  |  |  |  |  |  |  |  |
| 63.98 | 5.50 | 115.10 | 0.00 | 8.25 | 2002.00 | 26.60 | 50.72 | 16.11 | 6.80 |
| 7.75 | 139.24 | 44.13 | 0.00 | 0.00 | 1954.12 | 29.91 | 52.36 | 10.79 | 2002.00 |
| 2002.00 | 470.45 | 110.13 | 186.12 | 21.25 | 14.78 | 13.89 | 21.13 | 54.21 | 3.99 |
| 269.05 | 12.50 | 67.66 | 3.75 | 0.00 | 7.68 | 158.85 | 7.98 | 5.17 | 1576.46 |
| 181.73 | 66.28 | 9.75 | 1463.97 | 2002.00 | 69.48 | 20.39 | 24.09 | 7.83 | 7.54 |

| IL-5 | (pg/ml) |  |  |  |  |  |  |  |  |
| --- | --- | --- | --- | --- | --- | --- | --- | --- | --- |
| n.s.^a^ |  |  |  |  |  |  |  |  |  |
| 49.29 | 8.84 | 1.34 | 4.55 | 0.80 | 2.75 | 0.88 | 0.38 | 11.25 | 10.00 |
| 28.89 | 489.57 | 199.32 | 201.43 | 14.46 | 11.50 | 8.75 | 9.75 | 6.75 | 18.00 |
| 74.95 | 3.75 | 0.27 | 0.54 | 4.29 | 25.75 | 1.75 | 1.25 | 1.25 | 12.75 |
| 46.63 | 15.70 | 2.81 | 3.05 | 1009.32 | 826.62 | 90.06 | 73.75 | 36.50 | 2.66 |
| 11.02 | 26.48 | 8.91 | 47.89 | 11.48 | 24.92 | 440.85 | 66.86 | 13.55 | 9.44 |
| HA |  |  |  |  |  |  |  |  |  |
| 17.34 | 11.72 | 6.09 | 5.16 | 3.05 | 27.82 | 15.24 | 137.09 | 1.69 | 3.63 |
| 8.67 | 19.45 | 0.94 | 0.00 | 571.49 | 91.23 | 34.33 | 102.14 | 35.53 | 11.85 |
| 49.65 | 199.31 | 1954.61 | 71.60 | 709.06 | 202.22 | 95.94 | 465.85 | 47.56 | 4.35 |
| 21.33 | 5.39 | 3.05 | 2002.00 | 142.56 | 624.08 | 897.70 | 84.52 | 596.55 | 20.32 |
| 22.50 | 0.00 | 3.28 | 59.48 | 206.45 | 84.19 | 54.54 | 69.83 | 49.73 | 3.39 |
| EBNA |  |  |  |  |  |  |  |  |  |
| 21.62 | 15.14 | 15.68 | 10.81 | 10.81 | 42.90 | 7.03 | 2002.00 | 33.34 | 17.84 |
| 8.65 | 49.58 | 13.51 | 5.41 | 8.11 | 15.14 | 689.58 | 12.97 | 5.41 | 159.19 |
| 20.00 | 16.76 | 14.59 | 82.54 | 87.87 | 5.95 | 4.86 | 10.27 | 5.95 | 4.32 |
| 8.65 | 8.65 | 35.25 | 8.65 | 15.68 | 23.78 | 62.00 | 4.86 | 5.95 | 10.27 |
| 258.75 | 524.96 | 15.14 | 139.08 | 5.41 | 7.57 | 5.41 | 10.27 | 8.65 | 4.32 |

^a^n.s., not stimulated (un-stimulated)
