# Supplementary material for: Estimating Point and Interval Frequency of Antigen-Specific CD4+ T Cells Based on Short In Vitro Expansion and Improved Poisson Distribution Analysis
Source: PLoS One. 2012 Aug 7;7(8):e42340. doi: 10.1371/journal.pone.0042340 (PMC3413706; doi:10.1371/journal.pone.0042340)
Supplement: Table S8 — Calculation of inactive wells for IFN-γ and IL-5 secretion at day 14 cultures in the Ag-stimulated wells for all donors (#11, #12, #13, #14, #15, #16, #17). (DOC) [file pone.0042340.s008.doc]

**Table S8**. Calculation of inactive wells for IFN-γ and IL-5 secretion at day 14 cultures in the Ag-stimulated wells

|  |  |  | |  |  |  |  |  |  |  |
| --- | --- | --- | --- | --- | --- | --- | --- | --- | --- | --- |
| Donor # |  | IFN-γ (pg/ml) | | |  |  | IL-5 (pg/ml) | |  |  |
|  |  | Threshold*^a^* | Inactive wells*^b^* | | |  | Threshold | Inactive wells | |  |
|  |  |  | HA | | EBNA |  |  | HA | EBNA |  |
| 11 |  | 52.10 | 4 | | 8 |  | 63.40 | 25 | 26 |  |
| 12 |  | 77.93 | 3 | | 6 |  | 46.63 | 24 | 25 |  |
| 13 |  | 26.43 | n.p.*^c^* | | 1 |  | n.p. | n.p. | n.p. |  |
| 14 |  | 36.36 | 18 | | 16 |  | 29.68 | 22 | 27 |  |
| 15 |  | 96.59 | 5 | | 11 |  | 300.43 | 8 | 20 |  |
| 16 |  | 12.38 | 5 | | 9 |  | 300.00 | 28 | 24 |  |
| 17 |  | 30.72 | 26 | | 27 |  | 117.11 | 44 | 43 |  |

*^a^*Calculated on the basis of the distribution of un-stimulated wells

*^b^*Within the Ag-stimulated wells

*^c^*n.p., not performed
